# More comprehensive proprioceptive stimulation of the hand amplifies its cortical processing

**DOI:** 10.1152/jn.00485.2021

**Published:** 2022-07-20

**Authors:** Maria Hakonen, Timo Nurmi, Jaakko Vallinoja, Julia Jaatela, Harri Piitulainen

**Affiliations:** ^1^Faculty of Sport and Health Sciences, University of Jyväskylä, Jyväskylä, Finland; ^2^Department of Neuroscience and Biomedical Engineering, Aalto University School of Science, Espoo, Finland; ^3^Aalto NeuroImaging, Magnetoencephalography Core, Aalto University School of Science, Espoo, Finland; ^4^Department of Radiology, Massachusetts General Hospital, Harvard Medical School, Boston, Massachusetts

**Keywords:** acceleration, corticokinematic coherence, magnetoencephalography, proprioception, sensorimotor cortex

## Abstract

Corticokinematic coherence (CKC) quantifies the phase coupling between limb kinematics and cortical neurophysiological signals reflecting proprioceptive feedback to the primary sensorimotor (SM1) cortex. We studied whether the CKC strength or cortical source location differs between proprioceptive stimulation (i.e., actuator-evoked movements) of right-hand digits (index, middle, ring, and little). Twenty-one volunteers participated in magnetoencephalography measurements during which three conditions were tested: *1*) simultaneous stimulation of all four fingers at the same frequency, *2*) stimulation of each finger separately at the same frequency, and *3*) simultaneous stimulation of the fingers at finger-specific frequencies. CKC was computed between MEG responses and accelerations of the fingers recorded with three-axis accelerometers. CKC was stronger (*P* < 0.003) for the simultaneous (0.52 ± 0.02) than separate (0.45 ± 0.02) stimulation at the same frequency. Furthermore, CKC was weaker (*P* < 0.03) for the simultaneous stimulation at the finger-specific frequencies (0.38 ± 0.02) than for the separate stimulation. CKC source locations of the fingers were concentrated in the hand region of the SM1 cortex and did not follow consistent finger-specific somatotopic order. Our results indicate that proprioceptive afference from the fingers is processed in partly overlapping cortical neuronal circuits, which was demonstrated by the modulation of the finger-specific CKC strengths due to proprioceptive afference arising from simultaneous stimulation of the other fingers of the same hand as well as overlapping cortical source locations. Finally, comprehensive simultaneous proprioceptive stimulation of the hand would optimize functional cortical mapping to pinpoint the hand region, e.g., prior brain surgery.

**NEW & NOTEWORTHY** Corticokinematic coherence (CKC) can be used to study cortical proprioceptive processing and localize proprioceptive hand representation. Our results indicate that proprioceptive stimulation delivered simultaneously at the same frequency to fingers (D2–D4) maximizes CKC strength allowing robust and fast localization of the human hand region in the sensorimotor cortex using MEG.

## Graphical abstract

**Figure:**
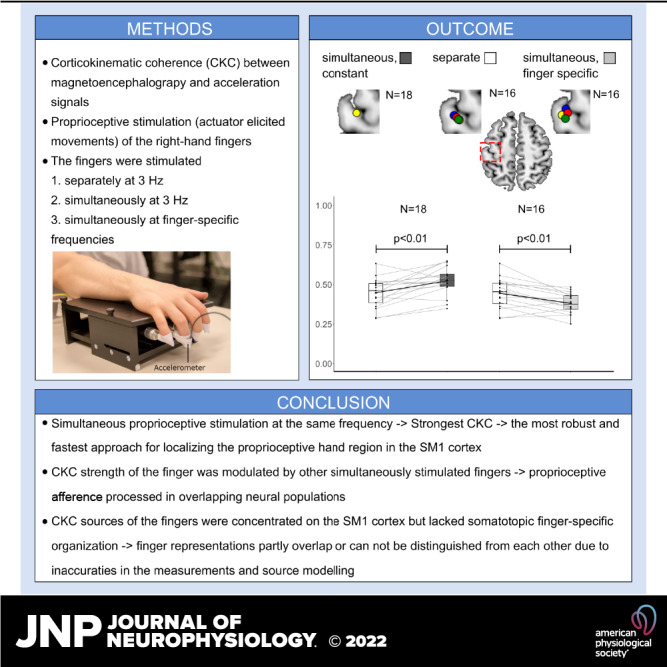

## INTRODUCTION

Corticokinematic coherence (CKC) quantifies the phase coupling between limb kinematics (e.g., hand acceleration or contractile force, 1) and cortical neurophysiological signals measured with magnetoencephalography (MEG) (2) or electroencephalography (EEG) in adults (3) and even in infants (4). CKC peaks at the movement frequency and its harmonics in the primary sensorimotor cortex (SM1) contralateral to the moving limb (5, 6). Active (volitional) and passive (evoked by an investigator) movements of the index finger elicit similar strength and cortical location of CKC (2, 7) suggesting that CKC reflects primarily somatosensory afference (presumably from the muscle spindles) to the SM1 cortex. CKC is strong for repetitive finger (2), toe (8), and ankle (9) movements and follows the respective somatotopic cortical representations. Moreover, it has shown to be reproducible at the group level both for MEG and EEG (3, 10). Thus, CKC is an attractive method to quantify the degree or extent of cortical proprioceptive processing and to pinpoint proprioceptive hand representation. Furthermore, CKC can provide complementary information to proprioceptive stimulation in fMRI for multimodal preoperative mapping when planning brain surgery (11). CKC may also be used to identify impairments in proprioceptive processing in various motor disorders (e.g., Refs. 12, 13) or in healthy aging (9).

Maximizing the degree of proprioceptive afference with a comprehensive proprioceptive stimulation of several fingers of the hand at the same time could potentially enhance the signal-to-noise ratio of the coherent cortical signal and better reflect the whole hand cortical representation than stimulation of one finger. Although the CKC is robust in healthy individuals, the comprehensive stimulation could be beneficial, especially when following the condition of certain clinical groups, such as patients with Friedreich’s Ataxia that have weaker CKC (14). It could also improve the sensitivity of the CKC to detect minute adaptations in cortical proprioceptive processing occurring, e.g., during healthy aging (9).

CKC studies of hand proprioception have usually stimulated the index finger (for a CKC study with volitional movements of D2–D5, see Ref. 15). Thus, it is unknown whether CKC differs between fingers and whether simultaneous movement of fingers is reflected in the CKC of the finger. Given that functional dominance and independence of the fingers vary (16–22), it can be hypothesized that this is also reflected as between-finger differences in CKC. Moreover, there is evidence that cortical representations of fingers partly overlap spatially, especially in motor (i.e., voluntary movements, 23–27) and also to some extent in tactile domain (28, 29). If the corresponding spatial overlap exists for proprioceptive representations, simultaneous movement of the other fingers may be reflected in the CKC of the finger. If this is true, it would be expected that simultaneous movement of the fingers at the same frequency would enhance CKC and, therefore, improve the robustness and time efficiency of CKC-based proprioceptive localization of the hand SM1 cortex. Alternatively, if CKC elicited by stimulation of the multiple fingers simultaneously is analogous to stimulating the finger separately, simultaneous stimulation of the fingers at finger-specific frequencies would provide a time-efficient CKC recording for the determination of cortical representations or CKC values of individual fingers (e.g., in specific clinical conditions).

Penfield and Boldrey (30) determined the sensory homunculus based on sensations produced by stimulation of the sensory cortex. In the sensory homunculus, the somatosensory representation of the index finger is the most dorsal and inferior one along the central sulcus followed by the representations of the middle and ring fingers and, finally, the most ventral and superior representation of the little finger. Although many studies have reported similar topographical organization of fingers using tactile stimuli with MEG (for five fingers: Refs. 31–33, for thumb and little fingers: Ref. 34, for thumb, index and little fingers: Ref. 35, for index and ring fingers: Ref. 36, for index and middle fingers: Ref. 37) and functional magnetic resonance imaging (fMRI) (25, 28, 38–46, 47), the corresponding proprioceptive representations are still largely unknown.

Only a few studies have directly compared the brain responses to proprioceptive and tactile stimuli using MEG (2, 48). Somatosensory-evoked magnetic fields to the two stimulus modalities are clearly separate in peak latencies, amplitudes, and orientations of equivalent current dipoles (48). It has also been shown that source localization of CKC and tactile stimulation differ for the index finger (2). Interestingly, CKC strength and source locations did not differ statistically between the conditions where the tip of the index finger was touching versus not touching the table, suggesting that cutaneous input did not substantially contribute to CKC.

The primary aim of this study was to examine whether the CKC strength or cortical source location differs between proprioceptive stimulation (i.e., movement actuator-evoked movements) of the right-hand digits (D2–D5: index, middle, ring, and little). We aimed to determine whether a comprehensive multifinger stimulation would improve the robustness and time efficiency of CKC-based proprioceptive localization of the hand SM1 cortex using MEG. Three conditions were tested: *1*) simultaneous stimulation of all four fingers at 3-Hz frequency (*simultaneous*_constant-ƒ_), *2*) stimulation of each finger separately at 3-Hz frequency (*separate*), and *3*) simultaneous stimulation of the fingers at finger-specific frequencies (at 2, 2.5, 3, and 3.5 Hz, *simultaneous*_varied-ƒ_).

We had four hypotheses. The first hypothesis (H1) was that the simultaneous stimulation of the four fingers would result in stronger CKC due to stronger proprioceptive afference to the SM1 cortex compared with the separate-finger stimulation. The second hypothesis (H2) was that CKC is weaker for *simultaneous*_varied-ƒ_ than *separate* condition because proprioceptive afference from the other simultaneously stimulated fingers distracts the finger acceleration phase-locking to MEG signals reducing the CKC strength. Third, we hypothesized (H3) that the CKC strength varies between fingers, which may reflect differences in functional dominance or independence of the fingers. Finally, the fourth hypothesis (H4) was that the cortical source locations of the proprioceptive responses of the fingers follow the finger-specific somatotopic order similarly as with tactile stimuli (31–37, 49).

## MATERIALS AND METHODS

### Participants

Twenty-one healthy Finnish participants (mean age: 27.8, SD: 4.9, range: 20–40, 10 females, mean handedness score: 77.1; SD: 41.3, range: –80–100, one left-handed, one ambidextrous) without neuropsychiatric diseases, movement disorders, or nonremovable metallic objects in their body volunteered in the study. The data of three participants were excluded from the comparisons of CKC strength or source locations between *simultaneous*_constant-ƒ_ and *separated* conditions and five between *simultaneous*_varied-ƒ_ and *separated* conditions because of bad signal quality. Thus, the total numbers of participants included in the final analyses were 18 (mean age: 27.5, SD: 5.2, range: 20–40, 8 females, mean handedness score: 75.2; SD: 44.1, range: –80–100, one left-handed, one ambidextrous) and 16 (mean age: 27.2, SD: 5.4, range: 20–40, 7 females, mean handedness score: 73.6; SD: 46.6, range: –80–100, one left-handed, one ambidextrous), respectively. The handedness scores were assessed by a modified Edinburgh handedness inventory (50). The study was approved by the ethics committee of Aalto University, and the participants gave written informed consent before participation in accordance with the Declaration of Helsinki.

### Experimental Design

**Figure 1.**
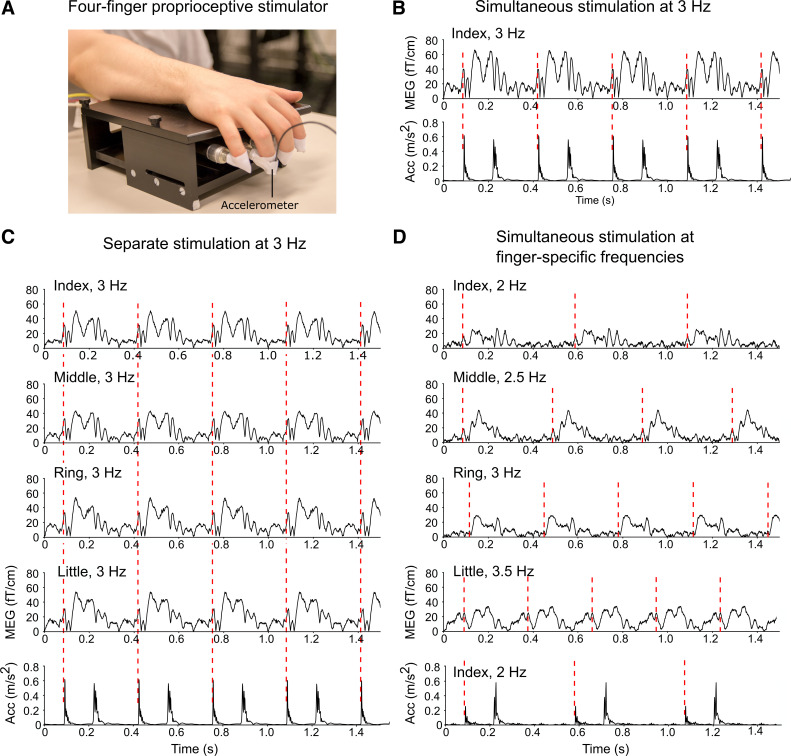
Proprioceptive stimulator, sustained-MEG fields for each finger, and acceleration magnitude for the index finger. *A*: the four-finger proprioceptive stimulator. Please note that the figure is only for visualization purposes and does not include all four accelerometers. *B–D*: averaged MEG responses (vector sum of the peak gradiometer pair) for each finger and acceleration magnitude (Euclidean norm of the three orthogonal components) for the index finger in all three conditions. The red dashed line indicates an onset of the flexion phase of the continuous flexion-extension movement. MEG, magnetoencephalography.

At the beginning of the MEG session, the participant was briefed about the experiment. Before entering the MEG, the participant was provided with nonmagnetic clothes and asked to remove any metallic objects he/she was wearing. During the MEG measurement, the participant was sitting with stimulated right hand on the custom-made proprioceptive stimulator (i.e., MEG-compatible movement actuator, Fig 1*A*) placed on the table. The stimulator was an extension of our previously developed one-finger stimulator (8). The tip of each of the four fingers was taped at the end of the finger-specific pneumatic muscle of the stimulator. In addition, a piece of surgical tape (Leukoplast) was lightly attached on the palmar surface of each fingertip to minimize tactile stimulation elicited by the tactile contact between the fingertips and the stimulator. The pneumatic muscles moved horizontally out of the stimulator straightening the proximal and slightly the distal interphalangeal joints of each finger. Accelerations of the fingers were measured with three-axis accelerometers (ADXL335 iMEMS Accelerometer, Analog Devices Inc., Norwood, MA) firmly taped on the nail of each finger. The left hand was resting on the thigh. The participant wore earplugs and Brownian noise was played in the background via a flat-panel speaker (Panphonics 60 × 60 SSHP, Tampere, Finland) to minimize auditory noise resulting from the airflow within the pneumatic muscles. To prevent the participant from seeing the moving fingers, a white A3-sized paper sheet was taped vertically to the MEG gantry. The participant was presented with a video of different landscapes (for two participants the video was not presented because of technical problems). Proprioceptive stimuli were controlled using Presentation software (v. 18.1, Neurobehavioral Systems, Albany, CA).

There were three conditions: *1*) simultaneous stimulation of all four fingers at 3 Hz (i.e., stimulus-onset asynchrony of 333 ms) in three 1-min bursts (*simultaneous*_constant-ƒ_, 3-min stimulation in total), *2*) stimulation of each finger separately at 3 Hz in three 1-min bursts (*separate*, 3-min stimulation per finger in total), and *3*) simultaneous continuous stimulation at finger-specific frequencies (given to D2, D3, D4, and D5 at 2, 2.5, 3 and 3.5 Hz, respectively; *simultaneous*_varied-ƒ_) for 4 min. The data for *simultaneous*_constant-ƒ_ and *separate* conditions were collected in the same measurement in 1-min runs (in total 3 min for each finger and *simultaneous*_constant-ƒ_). The stimulation order was pseudorandomized so that there were no consecutive stimulations to the same individual finger. The presentation order of the stimuli was the same for all subjects. The data for *simultaneous*_varied-f_ condition were collected in a separate measurement. Horizontal movement range of the pneumatic muscle was 3.2–4.7 mm depending on the condition (∼3 mm for *simultaneous*_constant-ƒ_ and *simultaneous*_varied-ƒ_, and ∼4.6 mm for *separate*; the movement range slightly differed between the conditions due to technical reasons related to the pneumatic stimulator, for more detailed discussion, see *Further Perspectives and Limitations*). The duration of this measurement was 15 min. Figure 1 shows the movement actuator as well as averaged MEG and acceleration signals measured during proprioceptive stimulation for a representative participant in three experimental conditions.

The finger-movement stimuli were within normal frequency of human hand actions and, thus, sensed as naturalistic continuous movements, although the movement range was relatively small. The proprioceptors are extremely sensitive even to subtle movements. For example, muscle spindles are sensitive to length changes as low as 5 µm during vibration of their parent muscle (51). In addition, joint receptors may have an important role in CKC. However, these receptors are known to be best activated near the end of the range of motion for a given joint and remain “silent” at the middle portion of the range of motion. The current movements occurred at neutral mid-portion of the finger range of motion. Thus, the role of muscle spindles may be emphasized as they are well activated throughout the range of motion. The movements were smooth and not brisk enough to evoke an overt stretch reflex in our healthy participants who did not report problems remaining passive during the stimulation. However, we did not record electromyographic activity to exclude the possibility of some mild muscle activation.

### Data Acquisition

MEG recordings were performed in a three-layer µ-metal magnetically shielded room (Imedco AG, Hägendorf, Switzerland) at MEG core of Aalto Neuroimaging Infrastructure (ANI) using a whole scalp MEG device (Vectorview 4-D Neuromag Oy, Finland), with 204 gradiometer and 102 magnetometer sensors. MEG signals were band-pass filtered at 0.1–330 Hz and sampled at 1 kHz. Eye blinks were detected from electro-oculography (EOG) signal using an electrode pair placed above and below the left eye. Five head-position indicator (HPI) coils were used to determine the position of the head with respect to the MEG sensors and to record head position continuously during the MEG recording. Before the MEG measurements, the locations of the HPI coils were recorded with respect to three anatomical landmarks (nasion and two preauricular points) using a three-dimensional digitizer (Isotrack, Polhemus, Colchester, VT). In addition, points on the scalp surface (∼100) were digitized to facilitate coregistration between MEG data and anatomical magnetic resonance images. During the measurement, the participants were in a seated position, and they were instructed to remain stationary and avoid blinking. The acceleration signals measured by the accelerometers attached on the nail of each finger were low-pass filtered at 330 Hz and sampled at 1 kHz time-locked to MEG signals.

Anatomical magnetic resonance imaging (MRI) images were acquired using a 3-tesla MRI scanner (MAGNETOM Skyra, Siemens Healthcare, Erlangen, Germany) and a 32-channel receiving head coil at the Advanced Magnetic Imaging (AMI) Center of Aalto University. MRI data were measured with a high-resolution T1-weighted magnetization-prepared rapid gradient echo (MPRAGE) pulse sequence [repetition time (TR) = 2,530 ms, echo time (TE) = 3.3 ms, flip angle = 7, 256 × 256 matrix, 176 sagittal slices, 1-mm resolution].

### MEG Preprocessing

MEG data were first visually inspected to identify noisy channels. Next, the uncorrelated sensor noise was reduced using the oversampled temporal projection (OTP, 52) algorithm. The temporally extended signal space separation algorithm [tSSS, MaxFilter 2.2 software, Elekta Neuromag Oy, Helsinki, Finland, (53), buffer length: 16 s, correlation limit: 0.95] was applied to the MEG data to reduce environmental magnetic noise and interpolate the noisy channels. Visually identified noisy channels were given as an argument to the OTP and tSSS algorithms, and an automatic noisy channel detection (autobad option) was used in tSSS to further identify any noisy channels. To remove eye blinks and heartbeats from the MEG signals, the data were decomposed into 30 independent components using fast independent component analysis (FastICA, 54). Independent components related to the blinks and heartbeats were identified by visually inspecting the topographies and time series of ICA components and, thereafter, subtracted from the data. The ICA components were determined from the data filtered between 1 and 40 Hz using a zero-phase finite impulse response filter (firwin in SciPy 1.2.1; Hamming window, 55) and removed from the nonfiltered data. OTP and ICA were performed using MNE Python software (v. 3.6, 56, 57). The acceleration data of four accepted participants were missing and, therefore, replaced with the accelerometer data from another participant (stimulus sequence was identical across participants). For four participants, the CKC was computed using another representative participant’s acceleration as a reference signal. This was appropriate in the current experiment as the stimuli were identical and time-locked with ms accuracy between the participants and because the signal-to-noise ratio of acceleration is excellent. It is noteworthy, that any possible small between-subject differences in the shape of the acceleration do not affect CKC strength, only the variation in stimulation onsets would reduce CKC strength.

### Sensor Level CKC Analysis

To compute CKC between MEG and accelerometer signals for each finger, continuous data were split into epochs of 4,000 ms with an overlap of 5 ms (5, 58). The MEG epochs exceeding 2,000 fT/cm at gradiometers and 4,000 fT at magnetometers in peak-to-peak amplitude were excluded automatically from the data. Acceleration corresponding to each MEG epoch was computed as Euclidean norm of the three orthogonal accelerometer signals band-passed between 0.5 and 195 Hz. The acceleration epochs were normalized by their Euclidean norms (5). Thereafter, power spectra were computed for each epoch as follows:

$$P_{xx}(f)=\frac{1}{K}\sum_{k=1}^{K}X_{k}(f)X_{k}^{\text{*}}(f)$$

$$P_{yy}\left(f\right)=\frac{1}{K}\sum_{k=1}^{K}Y_{k}(f)Y_{k}^{\text{*}}(f)$$

and cross-spectrum as:

$$P_{xy}(f)=\frac{1}{K}\sum_{k=1}^{K}X_{k}(f)Y_{k}^{\text{*}}(f)$$

where *X_k_*(*f*) and *Y_k_*(*f*) are the Fourier transformations of the *k^th^* MEG and acceleration epochs, respectively, and *K* is the number or epochs. CKC was then computed as follows (59):

$$Coh_{xy}\left(f\right)=\frac{{\left|P_{xy}(f)\right|}^{2}}{P_{xx}(f)P_{yy}(f)}.$$

Peak CKC strength was determined as the maximum coherence at the stimulation frequency over all MEG channels for each participant. The topographic distributions of CKC were visualized using Fieldtrip software (60). The threshold for statistical significance corrected for multiple comparisons was computed as follows separately for all fingers and conditions for each participant with the following equation:

$$coh_{thr}=1-{\left(\frac{0.05/N_{sens}}{N_{f}}\right)}^{\frac{1}{\left(N_{trials}/d-1\right)}}$$

where *N_sens_* is the number of MEG sensors among which the maximum coherence was searched, *N_f_* is the number of the frequencies of interest (i.e., one as we studies only the movement frequency), *N_trials_* number of trials, and *d* the overlap between trials (i.e., 5 ms, 58).

### Source Level CKC Analysis

Source modeling was used to estimate the CKC source locations and CKC strength. Source modeling typically yields a higher signal-to-noise ratio than the sensor-level analysis as spatial filtering suppresses irrelevant background activity. In addition, in the source space, all MEG sensors contribute to the CKC estimate, whereas in the sensor space, CKC is only estimated at the gradiometer pair showing the maximum CKC value.

The dynamic imaging of coherent sources (DICS) beamformer (5, 11, 61) was used to estimate CKC between MEG signals and Euclidian norm of the accelerometer signals in the source space. To this end, cortical surfaces were reconstructed from T1 images using FreeSurfer’s recon-all algorithm (Freesurfer software v. 6.0; 62, 63). To compute the forward model, a single-compartment boundary-element model (BEM) of the inner skull was generated using FreeSurfer’s watershed algorithm. Each participant’s MEG sensor positions and MRI data were coregistered by aligning fiducial points in MEG and MRI (i.e., nasion, left, and right preauricular points) as well as aligning MEG head digitization with the scalp. The fiducial points were manually identified on the MRI, and the fiducial registration error between MEG and MRI points was minimized by translating and rotating the MEG-digitized fiducials first automatically, and thereafter, adjusting the alignment manually. The forward model was computed for the volume source space with 3-mm spacing between the grid points. The leadfield with three components was reduced to the leadfield with two components corresponding to the highest singular values. The noise covariance matrix was estimated from the same file for which the source space CKC was computed. Finally, CKC maps were generated at the stimulation frequencies by computing CKC for all sources using DICS approach.

### Statistical Analyses

First, we investigated whether the strength of CKC differs between the simultaneous 3-Hz stimulation and separate 3-Hz stimulation (H1). To this end, a two-way 2 × 4 repeated measurements analysis of variance (rANOVA) was carried out, with the within-participant factors of condition (*simultaneous_constant-f_* vs. *separate*) and finger (index, middle, ring, and little). In addition, we computed CKC for the sum of the finger-specific MEG responses to separate stimulation (*separately_sum_*) and compared this to CKC for the simultaneous stimulation of the fingers at 3 Hz and separate stimulation (average CKC over fingers was used in *separate* condition). The main aim of this analysis was to see whether simultaneous stimulation at 3 Hz is analogous to the summation of the responses to the separately stimulated fingers. To this end, we conducted a one-way rANOVA with the within-participant factor of condition (*separate_sum_* vs. *simultaneous_constant-f_* vs. *separate*). Second, a two-way 2 × 4 rANOVA was conducted (factors: condition: *simultaneous_varied-f_* vs. *separate*; finger*:* index, middle, ring, and little) to study whether CKC strength differs between the simultaneous stimulation at the finger-specific frequencies and separate 3-Hz stimulation (H2). For each finger, the number of accepted trials in separate stimulation was set as an upper limit of the trials in simultaneous stimulation at the finger-specific frequencies as the MEG measurement under separate stimulation was 1 min shorter. Third, the main effect, finger, in the two-way 2 × 4 rANOVAs above was used to test the hypothesis that CKC is finger-specific (H3). We performed all rANOVAs separately for sensor and source level CKC strengths. If there were significant main effects or interactions in rANOVAs, Newman–Keuls post hoc test was used to determine which groups are different from each other. When finger-specific CKC values in separate stimulation were compared with the CKC of simultaneous stimulation at 3 Hz (i.e., one value), *P* values computed with Newman–Keuls post hoc test were further corrected using Benjamin–Yekutieli method (64, 65). Kolmogorov–Smirnov test and Mauchly test were run to test the normality and sphericity of the data, respectively. rANOVAs, Kolmogorov–Smirnov tests, and Mauchly tests were implemented with Statistica 7.1 (StatSoft. Inc. 1984–2005).

Finally, we studied whether the source location of CKC differs between fingers (H4). To this end, individual CKC coordinates were transformed into a common Montreal Neurological Institute and Hospital (MNI) coordinate system and their mean value was computed across subjects. The statistical significance was assessed with a nonparametric permutation test (66). Specifically, a null distribution of 100,000 samples was created for each pair of fingers by computing the same Euclidean distance after permuting the CKC coordinates of the subjects between the fingers. The original Euclidean distance was compared to the resulting permutation distribution and considered statistically significant at *P* < 0.05. To test the consistency of the CKC location, we also computed the Euclidean distances of CKC source locations for each finger when stimulated separately versus simultaneously at finger-specific frequencies. The statistical significance was again assessed with the nonparametric permutation test. The null distribution of 100,000 samples was created by permuting the CKC coordinates of the subjects between the conditions.

## RESULTS

We included only successful recordings of participants with clear CKC topographies and statistically significant (*P* < 0.05) CKC peaking at the stimulation frequency in the final analysis (*n* = 16–18 participants depending on the condition). The assumptions of normality or sphericity were not violated in the data. CKC was stronger at the sensor than at the source level (average CKCs across conditions: 0.45 ± 0.03 vs. 0.61 ± 0.02). However, the sensor and source level results were replicated well with only some minor differences as expected. In the following, the CKC strengths are reported at the source level. The sensor-level results are described only if they differed from the source-level results and are otherwise included in the supplementary material (all Supplemental material is available at https://doi.org/10.6084/m9.figshare.20076065). At the source level, the maximum CKC value across the brain is reported. At the sensor level, the CKC strength was reported from the gradiometer pair with the strongest CKC value. There were no systematic between-finger or between-condition differences in the MEG gradiometer (sensor) pair in which CKC peaked. CKC peaked in MEG422-MEG423 gradiometer pair (50% of the cases) or in a gradiometer pair just adjacent to it.

### Stronger CKC to Simultaneous than Separate-Finger Stimulation at 3 Hz (H1)

**Figure 2.**
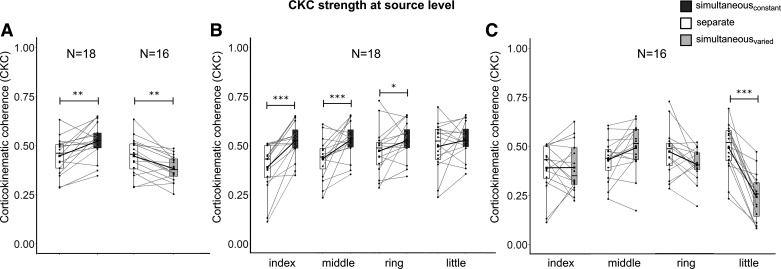
CKC strength for all conditions at the source level. *A*: average CKC strength for separate versus simultaneous stimulation at 3 Hz (*left*) and separate versus simultaneous stimulation at finger-specific frequencies. *B*: CKC strength for individual fingers when stimulated separately versus simultaneously at 3 Hz. *C*: CKC strength for individual fingers when stimulated separately versus simultaneously at finger-specific frequencies. Statistical differences were computed using a two-way repeated measurements analysis of variances.*P < 0.05, **P < 0.01, ***P < 0.001. CKC, corticokinematic coherence; *N*, number of subjects.

The total number of accepted trials did not differ significantly between the conditions (*simultaneous*_constant-ƒ_: 197 ± 21, *separate*: 199 ± 15, two-sample *t* test, *P* = 0.68, *n* = 18). Figure 2*A* and Table 1 present CKC strength for simultaneous and separate 3-Hz stimulation (for sensor-level results, see Supplemental Fig. S1*A* and Table S1). In line with our first hypothesis, CKC was stronger when the fingers were stimulated simultaneously than when they were stimulated separately, presumably reflecting stronger proprioceptive afference to the SM1 cortex. This effect was detected for all fingers expect the little finger at the source level and for all fingers at the sensor level. In addition, CKC obtained by using the average MEG responses across the four fingers when stimulated separately (0.81 ± 0.02) did not significantly differ from the simultaneous 3-Hz stimulation (0.78 ± 0.03) and was stronger than CKC for separate 3-Hz stimulation [0.56 ± 0.03, *P* < 0.001, main effect “condition”: *F*(2,34) = 19.9, *P* < 0.001, CKC values are reported as averages across subjects and fingers per condition; for description about the computation of CKC, see *Source Level CKC Analysis*). Thus, the CKC elicited by simultaneous stimulation at 3 Hz seems to be analogous to the CKC obtained for the summation of the finger-specific MEG responses elicited by separate stimulation.

**Table 1.** Group-averaged CKC strength for separate and simultaneous 3-Hz stimulations at the source level

H2 (*n* = 16)	Separate	Simultaneous_constant_	*P*	*F*	df1	df2
Finger average	0.45 ± 0.02	0.52 ± 0.02	**<0.003**	12.52	1	17
Interaction			**<0.002**	5.84	3	51
Index	0.40 ± 0.03	0.52 ± 0.02	**<0.001**			
Middle	0.43 ± 0.02	0.52 ± 0.02	**<0.001**			
Ring	0.47 ± 0.03	0.52 ± 0.02	**<0.029**			
Little	0.50 ± 0.03	0.52 ± 0.02	0.470			

Finger average is computed as an average over CKC values of all four fingers. Statistical differences were computed using two-way repeated measurements analysis of variances. CKC, corticokinematic coherence. *n*, number of subjects. Statistically significant (*P* < 0.05) *P* values are boldfaced.

### Weaker CKC to Simultaneous Stimulation at Finger-Specific Frequencies than Separate Stimulation at 3 Hz (H2)

The total number of accepted trials did not differ significantly between the conditions (*simultaneous*_varied-ƒ_: 199 ± 14 trials and *separate:* 199 ± 15, *n* = 16). In line with our second hypothesis, CKC was weaker when the fingers were stimulated simultaneously at the finger-specific frequencies (Fig. 2*A*, Table 2, and Supplemental Fig. S1*A* and Table S2), indicating that the simultaneous approach is not analogous with the separate finger stimulation. However, the reductions in CKC strength were finger-specific (Fig. 2*B*, Table 2, Supplemental Fig. S1*B* and Table S2). At the source level, CKC was only weaker for the little finger in the *simultaneous*_varied-ƒ_ condition. At the source level, CKC was weaker both for the little and ring fingers.

**Table 2.** Group-averaged CKC strength for separate 3-Hz stimulation and simultaneous stimulation at finger-specific frequencies at the source level

H2 (*n* = 16)	Separate	Simultaneous_varied_	*P*	*F*	df1	df2
Finger average	0.44 ± 0.02	0.38 ± 0.02	**<0.022**	13.67	1	15
Interaction			**<0.001**	22.71	3	45
Index	0.39 ± 0.03	0.39 ± 0.03	0.96			
Middle	0.43 ± 0.03	0.49 ± 0.03	0.089			
Ring	0.47 ± 0.03	0.40 ± 0.02	0.066			
Little	0.49 ± 0.03	0.24 ± 0.03	**<0.001**			

Finger average is computed as an average over CKC values of all four fingers. Statistical differences were computed using two-way repeated measurements analysis of variances. CKC, corticokinematic coherence. *n*, number of subjects. Statistically significant (*P* < 0.05) *P* values are boldfaced.

### CKC Strength Varied between Fingers (H3)

**Figure 3.**
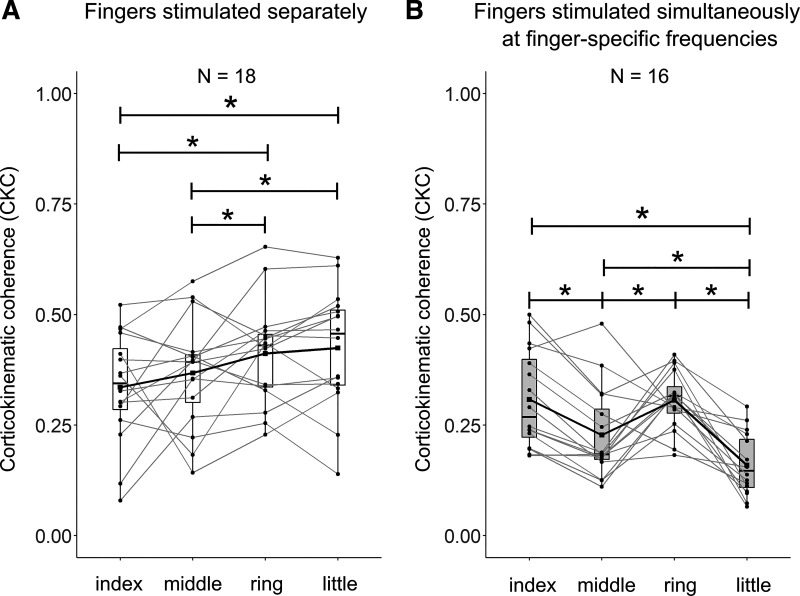
CKC strength for individual fingers at the source level. Average CKC for the separate stimulation of individual fingers (*A*) and for the simultaneous stimulation at the fingers at their specific frequencies (*B*). Statistical differences were computed using a two-way repeated measurements analysis of variances. *P < 0.05. CKC, corticokinematic coherence; *N*, number of participants.

Figure 3 shows the CKC strength for individual fingers elicited by separate stimulation (*A*) and simultaneous stimulation at finger-specific frequencies (*B*). CKC strength differed between the fingers (main effect *finger*: *F*(3,45) = 4.80, *P* < 0.01) and the relative between-finger differences were depending on the conditions (interaction *condition* × *finger*: *F*(3,45) = 22.7, *P* < 0.001). When stimulated separately, CKC was stronger for the little (0.50 ± 0.03) than the middle finger (0.43 ± 0.02, *P* < 0.02, Fig. 3*A*). The results were replicated at the sensor level (Supplemental Fig. S2*A*).

When the fingers were stimulated simultaneously at the finger-specific frequencies, CKC was stronger for the middle (0.49 ± 0.03) than index (0.39 ± 0.03, *P* < 0.01), ring (0.34 ± 0.02, *P* < 0.02), and little (0.24 ± 0.03, *P* < 0.001) fingers. Moreover, CKC was stronger for the index (*p* < 0.001) and ring (*p* < 0.001) fingers than for the little finger. The results were similar at the sensor level with the exception that in simultaneous stimulation at finger-specific, the only statistically significant difference was stronger CKC for the middle than the little finger (Supplemental Fig. S2*B*).

### CKC Source Locations Were Concentrated on Hand Region of the SM1 Cortex (H4)

**Figure 4.**
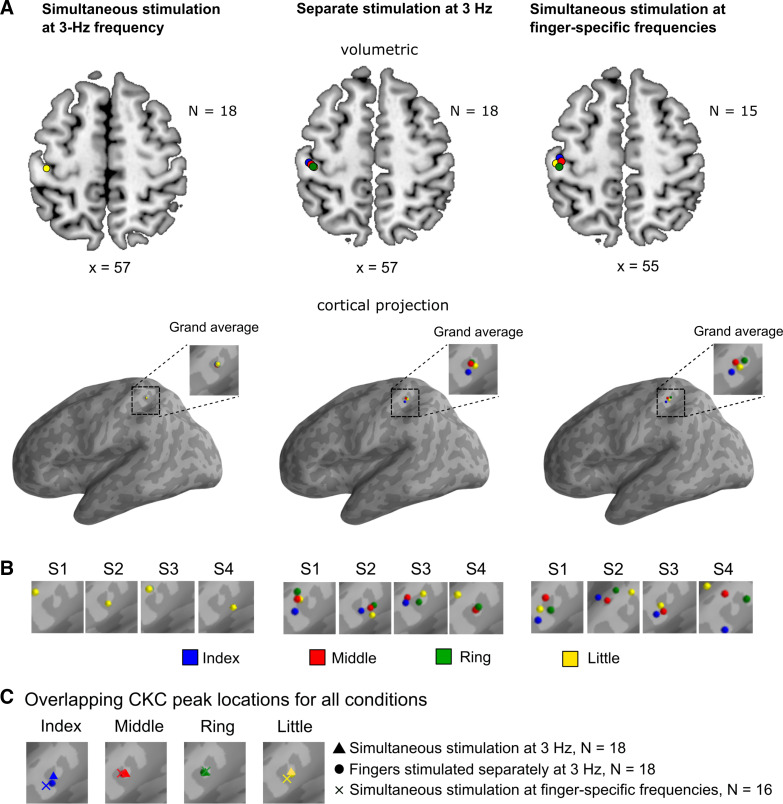
CKC-source-peak locations. *A*: group-level CKC source locations of each finger overlaid on the same volumetric brain (*top*) and cortical surface (*bottom*) separately for each condition. Please note that *x*-coordinates are averages over the x-directional MNI coordinates of the CKC source locations of the four fingers (for the MNI source coordinates of each finger, see Table 3). *B*: CKC source locations of each finger of four representative participants (S1–S4) overlaid on the same cortical surface separately for each condition. *C*: group-level CKC source locations of each condition overlaid on the same cortical surface separately for each finger. Please note that the source locations were concentrated on the Rolandic hand region of the SM1 cortex (i.e., central sulcus) in the source volume, but were misleadingly projected away from the central sulcus in the anterior wall of the postcentral sulcus when visualized to the cortical surface. CKC, corticokinematic coherence; *N*, number of participants.

In contrast to our fourth hypothesis, the source locations of the fingers were not distinct and did not follow the consistent finger-specific somatotopic pattern (Fig. 4 and Table 3) indicated by Penfield’s homunculus (30, 67) and subsequent studies with tactile stimuli (49, 68). CKC locations did not significantly differ between separate stimulation and simultaneous stimulation at finger-specific frequencies.

**Table 3.** The grand average MNI coordinates of CKC peak source locations

	Simultaneous_constant-ƒ_ (*n* = 18)			Separate (*n* = 18)			Simultaneous_varied-ƒ_ (*n* = 16)		
Finger	X, mm	Y, mm	Z, mm	X, mm	Y, mm	Z, mm	X, mm	Y, mm	Z, mm
Index	–43.4 ± 1.1	–23.9 ± 1.7	56.4 ± 1.1	–46.7 ± 0.9	–23.0 ± 1.8	52.9 ± 1.6	–46.2 ± 1.6	–19.4 ± 2.0	52.9 ± 1.5
Middle	–43.3 ± 1.1	–23.9 ± 1.7	56.3 ± 1.1	–45.2 ± 1.2	–24.2 ± 1.5	56.9 ± 1.4	–44.8 ± 1.6	–21.2 ± 2.0	57.3 ± 1.3
Ring	–43.5 ± 1.1	–24.1 ± 1.6	56.4 ± 1.1	–43.7 ± 0.8	–24.8 ± 1.6	57.7 ± 1.5	–46.6 ± 1.3	–25.0 ± 1.6	57.9 ± 1.8
Little	–43.4 ± 1.0	–24.0 ± 1.6	56.4 ± 1.1	–43.4 ± 1.1	–26.2 ± 1.4	56.1 ± 1.7	–48.5 ± 1.4	–22.9 ± 2.0	54.8 ± 2.0

Notice that there is only one CKC peak in simultaneous_constant_ condition since all fingers were moved at the same time with constant frequency. CKC, corticokinematic coherence; *n*, number of participants.

## DISCUSSION

We examined the CKC strength and cortical source location to proprioceptive stimulation of the right-hand fingers. Our results indicated that the strongest CKC was obtained with the most comprehensive stimulation of all four right-hand fingers at the same 3-Hz frequency. The simultaneous stimulation at 3 Hz resulted in ∼15% stronger CKC than stimulation the fingers separately. CKC was weakest for the simultaneous stimulation of the fingers at finger-specific frequencies (2–3.5 Hz), being ∼14% weaker than the CKC obtained with stimulation of the fingers separately and ∼37% weaker than the strongest CKC. The CKC was weaker for the middle than the little finger during separate stimulation, but the opposite was true during simultaneous stimulation at finger-specific frequencies, tentatively suggesting the functional dominance of the most independent fingers in complex multidigit movements. All CKC source locations were concentrated in the Rolandic hand region of the SM1 cortex with some differences but without a consistent finger-specific somatotopic order between the fingers. The simultaneous stimulation of all or several fingers can be suggested to improve robustness (signal-to-noise ratio) and time efficiency of proprioceptive localization of the SM1 cortex of the hand when using the CKC method in combination with MEG.

### Stronger CKC to Simultaneous than Separate-Finger Stimulation at 3 Hz

In agreement with our hypothesis, CKC was 15% stronger for the 3-Hz simultaneous simulation of the fingers (index, middle, ring, and little) than for the 3-Hz stimulation of the fingers separately. Our result extends previous studies that have stimulated the proprioceptors related to the index finger (3, 8, 9, 69). We showed that the coherent proprioceptive afference from all induced fingers sum up to the SM1 cortex proprioceptive processing. Thus, the more comprehensive is the proprioceptive afference, the stronger is the cortical response or related proprioceptive processing. The stronger CKC may therefore reflect multidigit converging of the proprioceptive input. Similarly, the efferent motor output from the motor cortex converges and diverges when activating the hand muscles (70, 71). Moreover, the fingers of the hand are functionally and anatomically overlapping. For example, activations of different neuromuscular regions in the monkey flexor digitorum profundus muscle have shown to produce uniquely distributed tension in all five digits (72). Thus, the neural control of the hand is likely more optimized for synergistic movements by combinations of fingers rather than control of individual fingers. Therefore, it is likely that the cortical proprioceptive processing is better optimized for the collective hand than individual digit movements. This hypothesis is further supported by a fMRI study that revealed that hand postural information, encoded through kinematic synergies of the fingers, strongly correlated with BOLD activation patterns in the SM1 cortex (73). An additional reason for the stronger CKC for the simultaneous finger stimulation could be the insufficient specificity of MEG to perfectly register the individual finger responses. MEG is biased toward neuronal activity from tangential currents, thus recording activity predominantly from sulci (i.e., fissural cortex) rather than gyri (74).

### Weaker CKC to Simultaneous Stimulation at Finger-Specific Frequencies than Separate Stimulation

In agreement with our hypothesis, CKC was 14% weaker for simultaneous stimulation at finger-specific frequencies than for the separate stimulation. Given that CKC has shown to be unaffected by the movement frequency of the finger (8, 15), it seems that proprioceptive afference from the other simultaneously stimulated fingers distracts the finger acceleration phase-locking to MEG signals reducing the CKC strength. As the fingers were stimulated with different frequencies, it is likely that the respective cortical responses are temporally overlapping in random manner, which likely hinders the respective signal-to-noise ratios and prominence of the MEG response (i.e., the coherent event), and eventually the CKC strength. Related result has been obtained in a study that showed stronger CKC between MEG and index finger acceleration for constant than jittered proprioceptive stimulation (75).

The reduction in CKC from separate to simultaneous stimulation was observed only for the little (∼51% weaker CKC) finger at the source space. At the senor space, CKC reduced both for the little and ring (∼26–29%) fingers. This observation may be due to their lower level of motor performance and independence, and thus the extent of the neuronal circuit responsible for the cortical proprioceptive processing for these fingers. It can also be hypothesized that during the simultaneous stimulation, the MEG signal is more dominated by the more independent fingers with higher motor performance, as the index and middle fingers, which may have larger cortical neuronal population involved in their proprioceptive processing. However, it should be noted that there is no previous evidence for this kind of functional principle and, therefore, more studies are needed to support this hypothesis. Another aspect is that cortical processing may partly overlap in the functionally closely related fingers of the same hand. The functional interconnections between fingers have been estimated by measuring finger kinematics and kinetics when the subject has been instructed to produce isolated one-finger contraction (20) or repetitive tapping (16, 17). Involuntary forces or movements by the noninstructed fingers were interpreted to reflect structural (i.e., tendons and muscles) and/or neuronal connections between the fingers. According to these studies, the index finger was the most independent and the ring finger the least independent of the other fingers. The middle finger was reported to be more independent than the little finger (16, 20) or vice versa (17). Similar results have been obtained when the independence of the finger has been estimated based on the degree of how well the kinetics of the other fingers predict the finger kinematics in everyday-hand movements (18). These results agree with our finding that the CKC strength of the most independent index finger was least affected by the simultaneous movement of the other fingers. Finally, based on our results, it appears that the level of independence and functional overlap in the fingers’ kinematics and functions are evident also in the cortical level of proprioceptive processing.

### CKC Strength Varied between Fingers

In line with our hypothesis, CKC varied between fingers being stronger for the little than middle finger (by ∼12%) in separate stimulation. A possible explanation for the stronger CKC is weaker motor performance and/or level of usage of the little finger. The middle finger is more used, e.g., for grasping than the ring and little fingers (76). Our interpretation is supported by a study that found stronger CKC to reflect worse standing balance performance in older (66–73 yr) and younger (18–31 yr) adults (9). Similarly, movement-related cortical potentials in the SM1 cortex have shown to be stronger for novices than for motor-skilled subjects (77, 78). Together, these results support the neural efficiency hypothesis, where a smaller neuronal population is recruited with improved motor efficiency and precision (79). Our results may also reflect between-finger differences in construction, location, and the number of involved muscles as well as in tendons and connective tissues. Stimulation of the little finger may also engage the neighboring fingers due to its low independence resulting in more cortical recruitment and, therefore, stronger CKC.

An opposite association was obtained when the fingers were stimulated at the finger-specific frequencies simultaneously. CKC was strongest for the middle finger (20%–50% stronger than for the other fingers) and weakest for the little finger (30%–50% weaker than for the other fingers). These results further demonstrate that the phase-locking of the MEG response and individual finger kinematics is affected by the other fingers in a finger-specific manner. It could be tentatively hypothesized that the fingers with the highest motor performance dominate or “lead” the cortical proprioceptive processing during complex movement sequences of the hand. However, further studies are needed to determine whether proprioceptive information is processed with this kind of functional principle.

### CKC Source Locations Were Concentrated on the Hand Region of the SM1 Cortex

Our fourth hypothesis was that the cortical source locations of the fingers follow the consistent finger-specific somatotopic pattern indicated by Penfield’s homunculus (30, 67). In contrast with our hypothesis, the source locations of the fingers were spatially overlapping and did not follow somatotopy. However, according to our best knowledge, there is no prior evidence about proprioceptive representations of the same hand in the human SM1 cortex, although there is ample evidence about somatotopic finger organization in cutaneous tactile domain obtained both using MEG (31–37, 49) and fMRI (25, 28, 38–46, 47). MEG is biased toward neuronal activity in the sulci (i.e., fissural cortex) and is less sensitive to deep and radial currents (74, 80). It is possible that due to these methodological limitations of MEG, we were unable to define the consistent proprioceptive finger representations in the SM1 cortex. The result may also reflect that proprioceptive information from the fingers in the same hand is processed in spatially overlapping neuronal populations in the cortex. These proprioceptive neuronal populations may form functionally more coherent neuronal networks than the corresponding tactile neuronal populations, as the proprioception is functionally more directly related to the overall hand motor control.

The peak CKC locations were concentrated on the Rolandic SM1 cortex replicating previous results obtained by proprioceptive stimulation of the index finger (3, 8, 9, 69). However, the exact spatial coordinates for the CKC source have been reported previously only for passive index finger movements elicited by an experimenter (2), not by precise stimulator.

### Further Perspectives and Limitations

Our results have practical implications for the proprioceptive mapping of the hand area in the SM1 cortex using CKC. We suggest the simultaneous stimulation of several fingers at the same frequency to further improve robustness and time efficiency of CKC method for quantifying, mapping, and following cortical proprioceptive processing in the hand region of the SM1 cortex. If the CKC strength of the individual fingers is of interest, each of the fingers should be stimulated separately rather than simultaneously at the finger-specific frequencies. This is because the simultaneous stimulation at the finger-specific frequencies resulted in the weaker CKC and, therefore, the signal-to-noise ratio likely decreased compared with the stimulation of the fingers separately. Moreover, as the reduction in the CKC strength was finger-specific, the simultaneous stimulation with finger-specific frequencies can be less reliable approach to investigate the relative extent of the fingers’ proprioceptive processing in the SM1 cortex.

The proprioceptive stimulation of the fingers was generated with our neuroimaging compatible four-finger movement actuator which is an extension of the previous one-finger movement actuator (8). The actuator had a millisecond timing accuracy and stabile stimuli, and it did not produce any artifacts to MEG signals. Thus, it provides a robust and reliable neuroimaging compatible tool to locate and investigate multifinger proprioceptive afference to the SM1 cortex. The stimulator is suitable to study mechanisms of various motor disorders as it allows meaningful reproducible comparisons between controls and patients who might have impaired ability to perform active motor tasks. However, it should be noted that the proprioceptive processing in the SM1 cortex may differ between passive and active movements and therefore, the four-finger actuator can only be used to investigate the processing of passive component of proprioception. Passive movements together with motor imagery would correspond more closely to the active movements and may also be beneficial in the rehabilitation of neurological patients. Indeed, imagined movements have shown to engage the same sensorimotor mechanisms as active movements do (81–83).

The movement range varied up to 1.5 mm between proprioceptive stimulation conditions. This was inevitable during the *simultaneous*_varied-ƒ_ condition as the duration of electronic valve openings had to be varied (from 286 to 500 ms) according to the different stimulation frequencies. Thus, some of the pneumatic artificial muscles had more time to release the pressurized air and thus returned slightly closer to their resting length. The movement range was ∼1 mm higher for *separate* than *simultaneous*_constant-ƒ_ condition, because the pressurized air input was common for all stimulators, and thus the pressure level was slightly lower when all fingers were stimulated at the same time leading to the reduction in the movement range. However, it can be expected that the relatively small differences in the movement range have a negligible effect on CKC strength as the proprioceptors respond rather to dynamic than static changes in the muscle/joint position, even as low as 5 µm change in muscle length (51, 84). It would also be expected that the larger movement range results in stronger MEG response thus increasing CKC. However, CKC was stronger for simultaneous than (smaller range of motion) separate stimulation of the fingers. To obtain a more constant movement range across different frequencies in the pneumatic-proprioceptive stimulators, more sophisticated electronically controlled throttles would be required to accurately control the air speed dynamically. Thus, there is room to improve the controllability of the current MEG-compatible pneumatic-movement actuators.

Each of the fingers was stimulated with an identical pneumatic muscle providing the same finger displacement at least when the movement frequency has been the same. As the fingers differ in size, the actual range of motion in each metacarpophalangeal joint is indeed slightly different. However, it is unlikely that this small variation affects the CKC strength significantly as all fingers showed strong CKC and the proprioceptors are known to be primarily sensitive to the dynamic change. Thus, the actual change in the muscle length is likely less important for CKC strength, i.e., the proprioceptive afference is more related to the number of proprioceptors activated in the parent muscle of each finger than the degree of the movement evoked. This primarily anatomical limitation in the CKC experiments in MEG environment is hard to control, as it would need accurate real-time biomechanical data on the finger joint angles using, e.g., optical motion capture systems, which are not necessarily compatible with MEG.

The CKC was stronger when fingers were stimulated separately at 3 Hz than when they were stimulated simultaneously at finger-specific frequencies. The limitation in this design was that the fingers were stimulated at different frequencies although within a similar range (2–3.5 Hz). This is most likely a minor issue as the movement frequency of the finger has not been shown to affect the CKC strength (8, 15). Moreover, the CKC strength was stronger also for the ring finger in separate stimulation even though it was stimulated at 3 Hz in both conditions. Therefore, this effect was likely predominantly driven by the separate versus simultaneous condition difference but could be partly affected by the difference in the stimulating frequencies.

Finally, the passive movement actuator does not activate solely the proprioceptors but inevitably also the functionally closely related tactile mechanoreceptors of the skin that respond, e.g., to the stretch of the skin, and thus provide the brain with functionally overlapping information together with the proprioceptors. In future studies, simultaneous tactile stimulation of the fingertip during the movement could be used to assess whether the tactile afference contributes to the CKC strength in passive conditions. However, CKC strength has shown to be unaffected by the level of tactile stimulation of the fingertip during active and passive index-finger movements when quantified with MEG (2), and therefore, CKC primarily reflects cortical processing of proprioceptive afference at least in single-finger movements. With that being said, the tactile mechanoreceptors, responding, e.g., to stretch of the skin, can also be considered as a part of the same system providing the brain relevant functionally overlapping information about the peripheral movements and actions.

### Conclusions

The most comprehensive proprioceptive stimulation of the fingers simultaneously at the same frequency elicited the strongest CKC and can, therefore, be recommended as the most robust and fastest approach for localization of the human proprioceptive hand region in the SM1 cortex using MEG. The modulation of the CKC strength in an individual finger by the other simultaneously stimulated fingers suggests that the respective proprioceptive afference is being processed in partly overlapping cortical neuronal circuits or populations. CKC was stronger for the little finger than for the middle finger suggesting that functional dominance of the fingers may be reflected in CKC strength. An opposite observation was true when the fingers were stimulated simultaneously, which may reflect that the most dexterous fingers dominate the cortical proprioceptive processing. As expected, the CKC sources of the fingers were concentrated in the Rolandic hand region of the SM1 cortex, but surprisingly, lacked a systematic somatotopic finger-specific organization. Thus, the finger proprioceptive representations appear partly overlapping, and/or the nearby proprioceptive finger representations of the same hand cannot be separated adequately due to inaccuracies in measurements and source modeling.

## SUPPLEMENTAL DATA

10.6084/m9.figshare.20076065 Supplemental Figs. S1 and S2 and Supplemental Tables S1 and S2: https://doi.org/10.6084/m9.figshare.20076065.

## GRANTS

This study has been supported by the Academy of Finland (Grants Nos. 296240, 326988, 307250 and 327288, to H.P.), Jane and Aatos Erkko Foundation (to H.P.), “Brain changes across the life-span” profiling funding (311877, University of Jyväskylä), Finnish Cultural Foundation (to M.H.), and Paulo Foundation (to M.H.).

## DISCLOSURES

No conflicts of interest, financial or otherwise, are declared by the authors.

## AUTHOR CONTRIBUTIONS

T.N. and H.P. conceived and designed research; T.N. performed experiments; M.H., T.N., J.J., J.V., and H.P. analyzed data; M.H., T.N., and H.P. interpreted results of experiments; M.H. prepared figures; M.H. drafted manuscript; M.H., T.N., J.V., J.J., and H.P. edited and revised manuscript; M.H., T.N., J.V., J.J., and H.P. approved final version of manuscript.
